# Multi-Center Analysis of the Association Between Meteorological Factors and Aneurysmal Subarachnoid Hemorrhage Using Logistic and Machine Learning Models in Upstate New York

**DOI:** 10.3390/jcm15187314

**Published:** 2026-09-20

**Authors:** Avi A. Gajjar, Aditya D. Goyal, Ali Naqvi, Samhita Bheemireddy, Amanda Custozzo, Vinay Jaikumar, Adnan H. Siddiqui, Alan S. Boulos, John C. Dalfino, Alexandra R. Paul

**Affiliations:** 1 Department of Neurosurgery, Albany Medical Center, Albany, NY 12208, USA; 2 Department of Neurosurgery, University at Buffalo, Buffalo, NY 14203, USA

**Keywords:** intracranial aneurysm rupture, subarachnoid hemorrhage, meteorological factors, weather patterns, atmospheric pressure, UV index, seasonal variation, barometric pressure, cerebrovascular risk

## Abstract

**Introduction:** Ruptured intracranial aneurysms (RIAs) are a significant cause of morbidity and mortality. While individual-level risk factors for aneurysmal subarachnoid hemorrhage (aSAH) are well established, the influence of meteorological variables has been widely debated but remains unclear. **Methods:** We retrospectively analyzed 1504 endovascularly treated intracranial aneurysm cases from two stroke centers (2018 to 2024). We matched daily weather data to presentation dates. We used variance inflation factor (VIF) analysis to remove collinear features. Multivariable logistic regression models were adjusted for age and sex. We developed extreme gradient boosting (XGBoost) models using weather variables alone and in combination with demographic covariates (age, sex, race, smoking status, and family history). **Results:** Of 1504 cases, 377 (25.1%) presented with rupture. In univariate logistic regression, greater humidity (odds ratio [OR] 0.988 per 1% relative humidity, 95% confidence interval [CI] 0.979 to 0.998, *p* = 0.0135) and greater ultraviolet (UV) index (OR 0.951 per index unit, 95% CI 0.914 to 0.990, *p* = 0.0146) were associated with reduced odds of rupture, whereas greater snow depth (OR 1.360 per inch, 95% CI 1.068 to 1.731, *p* = 0.0126) and advanced moon phase (OR 1.589 per full lunar cycle, 95% CI 1.064 to 2.373, *p* = 0.0237) were associated with increased odds. On multivariable analysis, only female sex remained protective (OR 0.605, 95% CI 0.386 to 0.949, *p* = 0.0285), and greater sea level pressure trended toward lower odds of rupture without reaching significance (OR 0.962, *p* = 0.0523). Findings were consistent in a full-cohort, center-adjusted sensitivity analysis. The weather-only XGBoost model yielded an area under the receiver operating characteristic curve (AUC) of 0.560 and a recall of 78%, which improved to an AUC of 0.590 and a recall of 83% after adding demographic variables. SHapley Additive exPlanations (SHAP) analysis identified precipitation and cloud cover as key meteorological features. **Conclusions:** Several weather variables correlated with rupture risk in univariate analysis, but overall predictive value was limited. Machine learning improved sensitivity while confirming patient features as the dominant contributors. Weather on the day of presentation was not significantly associated with rupture.

## 1. Introduction

Intracranial aneurysm rupture remains a significant cause of morbidity and mortality, with aneurysmal subarachnoid hemorrhage (aSAH) having high fatality and disability rates. While well-established risk factors, such as aneurysm size, location, smoking, and hypertension, influence rupture risk [1], environmental factors, particularly weather patterns, have been weakly linked to cerebrovascular events [2]. Previous studies have suggested that variations in temperature, barometric pressure, and seasonal changes may influence the incidence of ischemic stroke and spontaneous intracerebral hemorrhage [3,4,5,6,7]. However, the relationship between weather patterns and aneurysmal rupture remains unclear, with conflicting findings and a lack of large-scale, multicenter investigations [8,9,10].

Anecdotal observations suggest that the frequency of ruptured aneurysms may vary by season, with an apparent increase during colder months. This raises the question of whether temperature fluctuations, humidity, atmospheric pressure, or other meteorological variables contribute to aneurysm rupture. Some studies have reported a higher incidence of aSAH in colder months, hypothesizing that vasoconstriction in response to lower temperatures could increase hemodynamic stress on the aneurysmal wall [11]. Cold exposure also increases blood viscosity and sympathoadrenal activation, transiently raising systemic blood pressure and shear stress on the aneurysm wall [4,11]. However, the evidence remains inconsistent, and small sample sizes and methodological variability have often limited previous investigations [5,7,11].

Given longstanding speculation that seasonal and environmental factors may influence aneurysm rupture risk, we sought to further investigate this relationship using contemporary clinical and meteorological data. The analysis was limited to Upstate New York, where two large comprehensive stroke centers with consistent reporting and reliable regional weather data provided an ideal setting for this investigation. This multicenter study therefore aims to determine whether environmental factors impact rupture risk.

## 2. Materials and Methods

### 2.1. Study Design and Setting

We conducted a retrospective, multicenter study to investigate the relationship between weather patterns and intracranial aneurysm rupture. Data were collected from January 2018 to November 2024. Patients were included if they had a confirmed diagnosis of an endovascularly treated intracranial aneurysm, with rupture status recorded at the time of admission. Cases with missing rupture information or incomplete date records were excluded. The primary outcome variable was aneurysm rupture, modeled as a binary outcome (ruptured vs. unruptured). Weather variables were extracted from Visual Crossing Weather Data and matched to the date of admission. We used the date of admission as the index date because it was the only temporal anchor consistently recorded across both institutional registries.

### 2.2. Weather Data

We obtained weather data from Visual Crossing Weather Data, a provider of historical meteorological information sourced from global observation stations, including the National Oceanic and Atmospheric Administration (NOAA) [12]. The dataset included temperature (maximum, minimum, mean, and “feels like” temperatures), humidity and precipitation (dew point, relative humidity, precipitation amount, probability, and coverage), as well as snow data (snowfall amount and snow depth). Additional meteorological variables included wind parameters (wind gust speed, wind speed, and wind direction), atmospheric pressure (sea level pressure), cloud cover and visibility (cloud cover percentage and visibility distance), and solar-related metrics (solar radiation and solar energy). The dataset also included ultraviolet (UV) index, severe weather risk, sunrise and sunset times, moon phase, and general weather conditions. We linked each patient to the corresponding daily weather data from the city where they were treated.

### 2.3. Statistical Analysis

**Figure 1:**
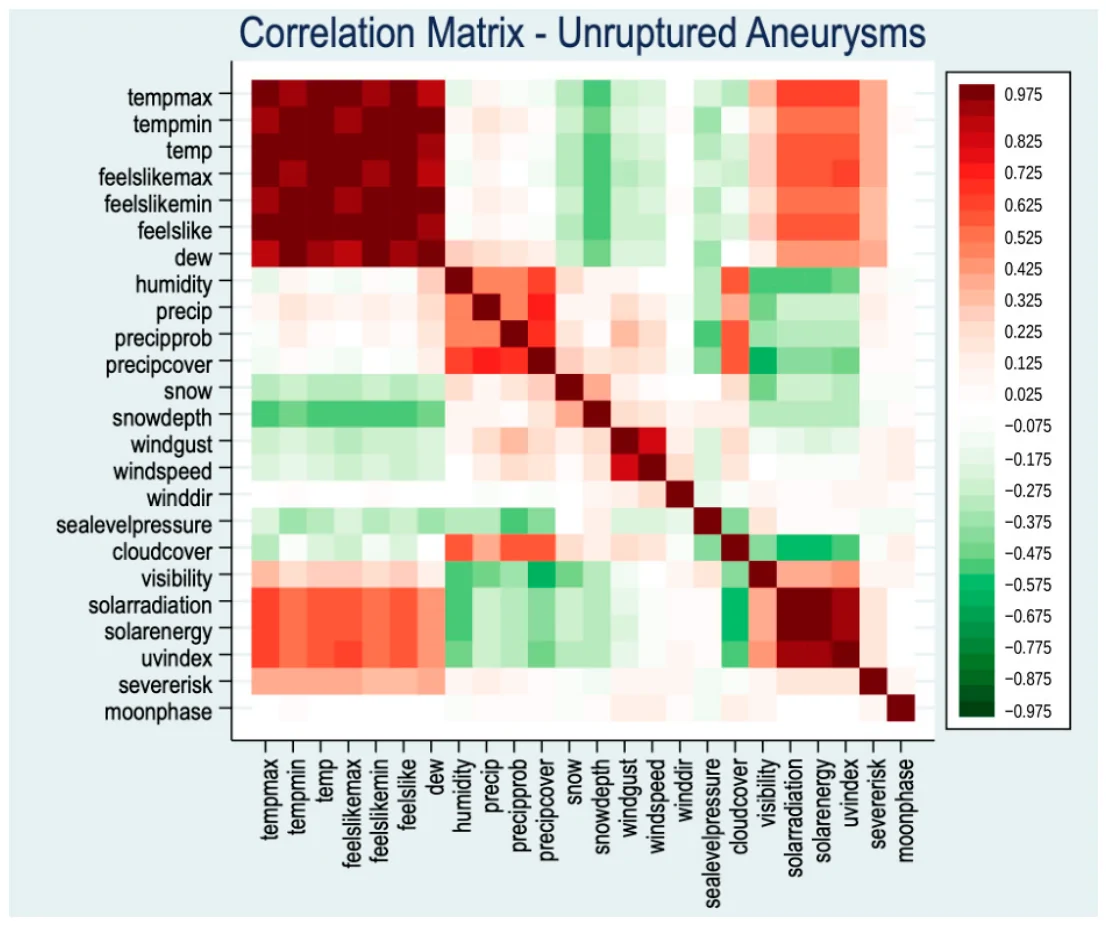
Correlation matrix for unruptured aneurysms.

**Figure 2:**
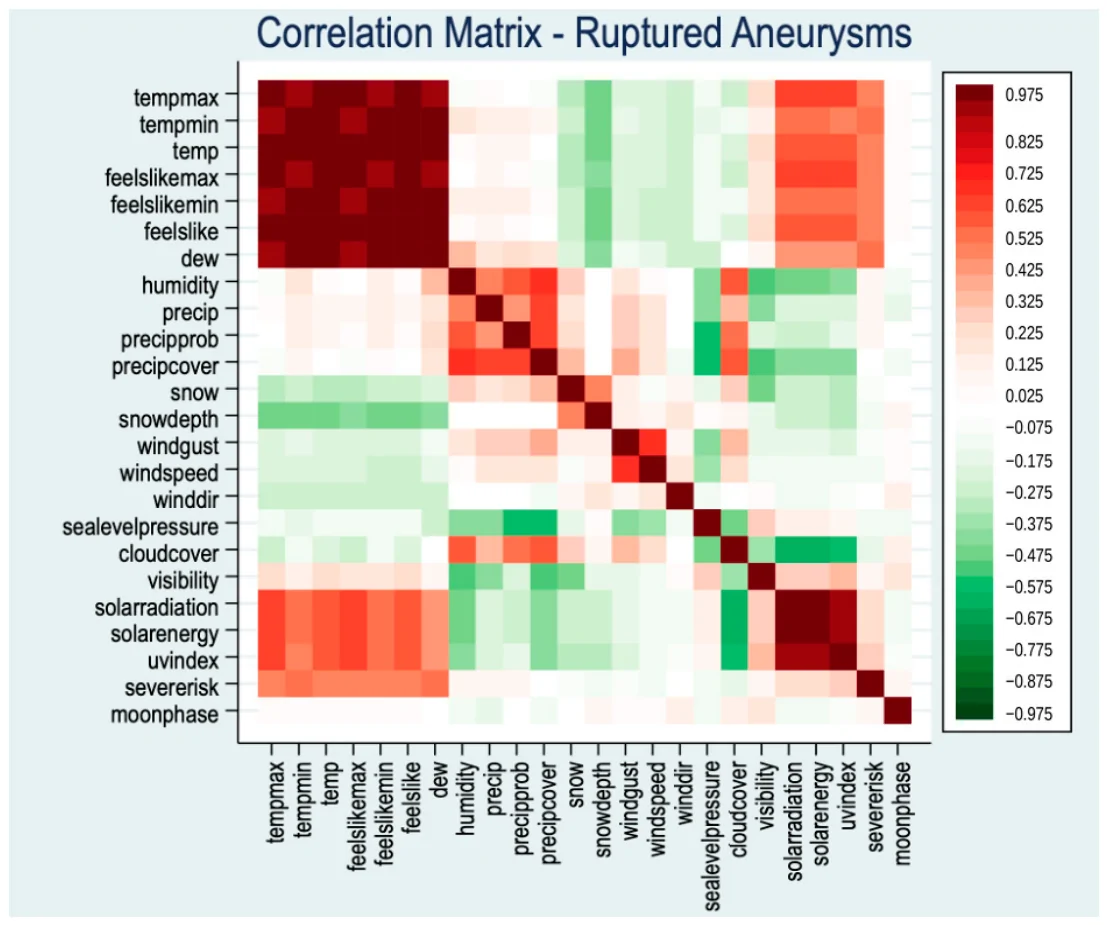
Correlation matrix for ruptured aneurysms.

A variance inflation factor (VIF) analysis was conducted via linear regression to reduce multicollinearity (Figure 1 and Figure 2). The VIF measures how strongly a predictor correlates with other independent variables in the model. A threshold of greater than 10 was used to identify severe collinearity, with affected variables removed from the final model. Variables that exceeded this threshold included solar radiation, solar energy, mean temperature, dew point, perceived temperature, minimum perceived temperature, minimum temperature, humidity, and maximum perceived temperature. These variables were excluded to improve model stability and avoid redundancy. Dew point demonstrated substantial collinearity with the other temperature-related variables, consistent with its strong correlation with other atmospheric measures. Maximum temperature was retained regardless of collinearity because of its established relevance in prior literature and potential biological plausibility in influencing rupture [13,14,15].

After removing collinear variables, the final predictive model included the following weather factors: maximum temperature, precipitation, probability of precipitation, precipitation coverage, snow accumulation, snow depth, wind gust speed, wind speed, wind direction, sea level pressure, cloud cover, visibility, UV index, severe weather risk, and moon phase. The model was adjusted for age and sex, both established risk factors for aneurysm rupture [16]. Race was evaluated in a sensitivity analysis but was not retained, as it did not significantly alter model estimates (χ^2^(4) = 9.12, *p* = 0.0581). In contrast, sex remained a significant independent predictor (χ^2^(1) = 5.50, *p* = 0.0190) and was included in all multivariable analyses.

A logistic regression model was constructed with aneurysm rupture as the outcome variable and the selected weather variables as predictors. Odds ratios (ORs) with 95% confidence intervals (CIs) were estimated to assess the association between weather patterns and rupture risk, with statistical significance defined as *p* < 0.05. ORs are reported per one-unit change in each predictor’s native measurement units. All statistical analyses were performed using Stata SE V18.0 (StataCorp, College Station, TX, USA).

### 2.4. Machine Learning Analysis for Nonlinear Relationships

To assess potential nonlinear associations between meteorological variables and aneurysm rupture, we implemented an extreme gradient boosting (XGBoost) model. XGBoost is a gradient boosting decision tree-based ensemble algorithm optimized for speed and performance, and it is well suited for imbalanced datasets where the majority class dominates [17]. Class imbalance occurs in cases where there is a majority class (unruptured aneurysm cases) that heavily dominates a minority class (ruptured aneurysm cases) in prevalence. To address this imbalance, we applied the scale_pos_weight parameter in XGBoost to mitigate bias toward the majority class.

Two models were then developed:

1. A weather-only model that included cloud cover, humidity, wind speed, UV index, moon phase, precipitation, and snow depth.
2. A composite model that incorporated the same meteorological variables alongside demographic covariates (age, sex, race, family history of aneurysm, and smoking status).

Variable selection for both models was guided by results from the univariate logistic regression and VIF screening. Variables with VIF > 10 were excluded as described previously to reduce feature redundancy, as collinearity filtering improves stability and interpretability even in tree-based models [18]. Humidity exceeded this threshold but was included in the weather-only machine learning model. Model performance was evaluated using the F2 score (to prioritize recall over precision), receiver operating characteristic (ROC) curves, and SHapley Additive exPlanations (SHAP) values to interpret feature contributions to model predictions.

For model interpretability, we applied SHAP summary plots to rank predictors by their average marginal contribution to model output. SHAP dependence plots were then generated for select meteorological and demographic variables, allowing visualization of each predictor’s direction and magnitude of effect, as well as potential effect modification by a third covariate. For these analyses, all other variables were overlaid using a red–blue gradient to identify interactions, consistent with established frameworks [19,20].

All machine learning analyses were conducted in R version 4.5.1 (GNU General Public License, R Foundation for Statistical Computing, Vienna, Austria). Figures were generated using R and Stata/SE version 18 (StataCorp LLC, College Station, TX, USA).

## 3. Results

### 3.1. Demographics

A total of 1504 endovascularly treated aneurysm cases were included in the analysis (Table 1), with 377 (25.1%) presenting with a ruptured aneurysm and 1127 (74.9%) with an unruptured aneurysm. The mean age was slightly lower in the ruptured group (57.7 ± 13.7 years) than in the unruptured group (59.5 ± 13.9 years, *p* = 0.0279). Females comprised most of the unruptured cohort (*n* = 867, 76.9%, *p* < 0.0001).

Hunt-Hess grade was available for 118 of 377 ruptured cases. Hypertension, blood pressure, and aneurysm size and location were not captured. Race, smoking status, and family history were recorded in the dataset assembled for the machine learning analyses and are therefore not included in Table 1.

### 3.2. VIF Analysis and Multivariable Logistic Regression Results

We first performed VIF analysis to evaluate collinearity across weather variables. In univariate logistic regression used for variable screening (Table 2), greater humidity (OR 0.988 per 1% relative humidity, 95% CI 0.979–0.998, *p* = 0.0135) and greater UV index (OR 0.951 per index unit, 95% CI 0.914–0.990, *p* = 0.0146) were associated with reduced odds of rupture. In contrast, greater snow depth (OR 1.360 per inch, 95% CI 1.068–1.731, *p* = 0.0126) and advanced moon phase (OR 1.589 per full lunar cycle, 95% CI 1.064–2.373, *p* = 0.0237) were positively associated with rupture. Notably, these univariate associations were used for variable screening and should not be interpreted as adjusted estimates. When evaluated in the multivariable logistic regression model, female sex was independently associated with lower odds of aneurysm rupture (OR 0.605, 95% CI 0.386–0.949, *p* = 0.0285). Greater sea level pressure trended toward a protective association against rupture (i.e., lower pressure trended toward greater odds of rupture), though this did not reach statistical significance (OR 0.962, 95% CI 0.926–1.000, *p* = 0.0523) (Table 3). In a full-cohort sensitivity analysis (*n* = 1504) excluding severe weather risk and adjusting for treatment center, female sex (OR 0.642, 95% CI 0.490–0.840, *p* = 0.0013) and age (OR 0.991, *p* = 0.0455) remained independently associated with rupture, treatment center was strongly associated with rupture status (OR 3.99, *p* < 0.0001), and no meteorological variable showed a robust association; only moon phase (*p* = 0.0496) and wind direction (*p* = 0.0195) reached nominal significance, and the sea level pressure trend did not persist.

In Table 2 and Table 3, ORs are per one-unit change in each variable’s native measurement units (temperature, feels-like temperature, and dew point in °F; humidity, precipitation probability, precipitation coverage, and cloud cover in percent; precipitation, snowfall, and snow depth in inches; wind gust and wind speed in mph; wind direction in degrees; sea level pressure in mb; solar radiation in W/m^2^; solar energy in MJ/m^2^; visibility in miles; moon phase on a 0 to 1 scale). Severe weather risk was reported only from 2022 onward; estimates involving it are based on 495 complete cases.

### 3.3. Model Performance

Model performance was evaluated using the Hosmer–Lemeshow goodness-of-fit test (χ^2^ = 6.63, *p* = 0.5769), which indicated good fit. Model testing classified cases as positive if the predicted probability of rupture was ≥0.5. Using rupture status as the reference standard, model sensitivity was 3.97% and specificity was 97.56%. The positive predictive value was 35.71%, and the negative predictive value was 74.84%. The false positive rate for actual unruptured cases was 2.44%, and the false negative rate for actual ruptured cases was 96.03%. Among classified positives, 64.29% were false positives, and among classified negatives, 25.16% were false negatives. Overall, 73.74% of cases were correctly classified. The model accurately identified most unruptured aneurysm cases, with a high specificity of 97.56%, but performed poorly in detecting ruptured aneurysms, with a sensitivity of only 3.97%. This indicates that it correctly classified only a small fraction of actual ruptures, despite the binary nature of the outcome.

### 3.4. XGBoost Results

The weather-only model showed limited overall predictive performance (area under the ROC curve [AUC] = 0.560, F2 = 0.598, accuracy = 47%), although recall was relatively high at 78%; specificity and precision remained low. The SHAP summary plot identified lower cloud cover, wind speed, and humidity as impactful nonlinear predictors of rupture risk, with both high and low values contributing variably to the model output. Feature importance analysis identified these same three variables as the most impactful factors included.

**Figure 3:**
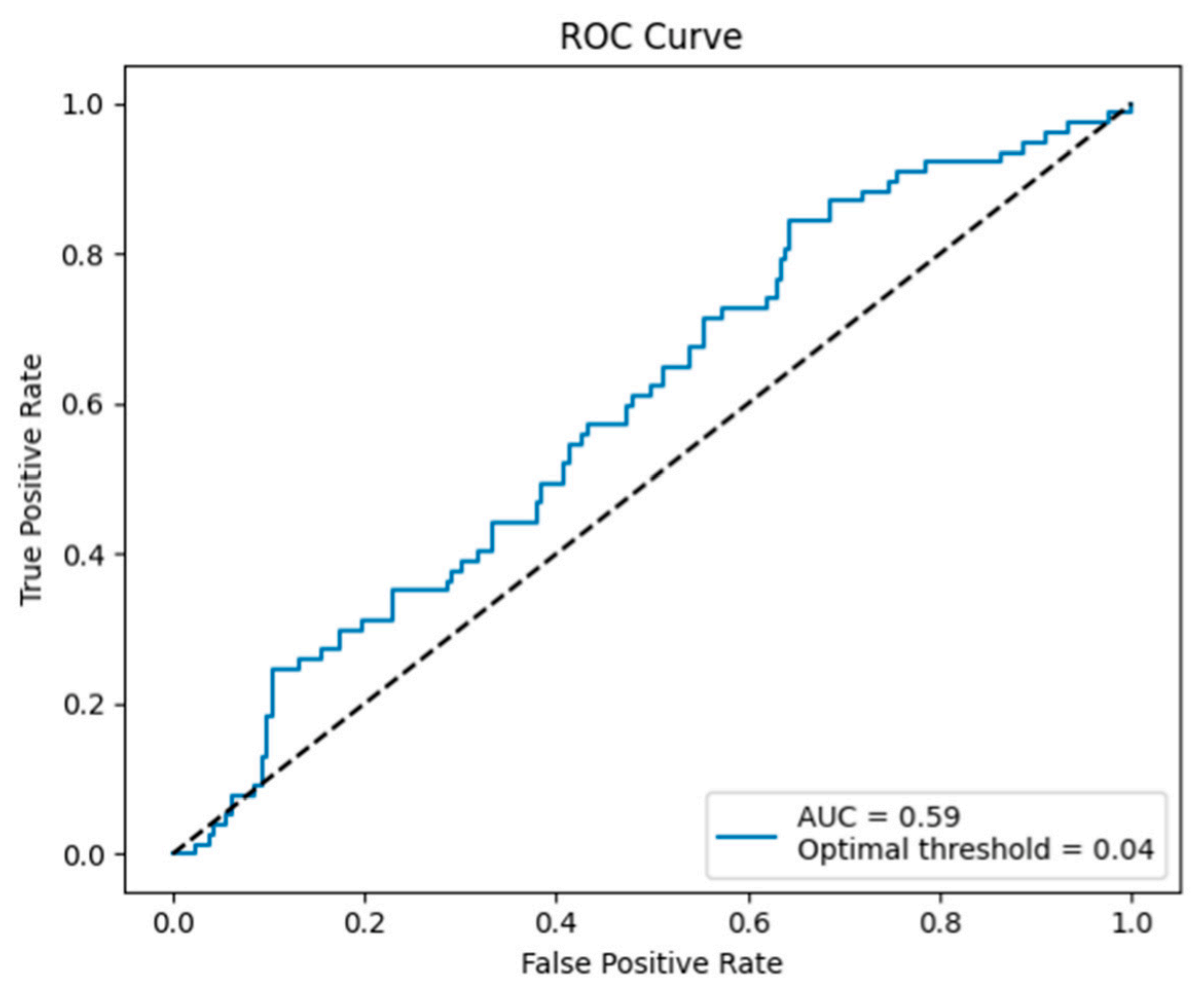
ROC curve for the machine learning model with demographic variables.

**Figure 4:**
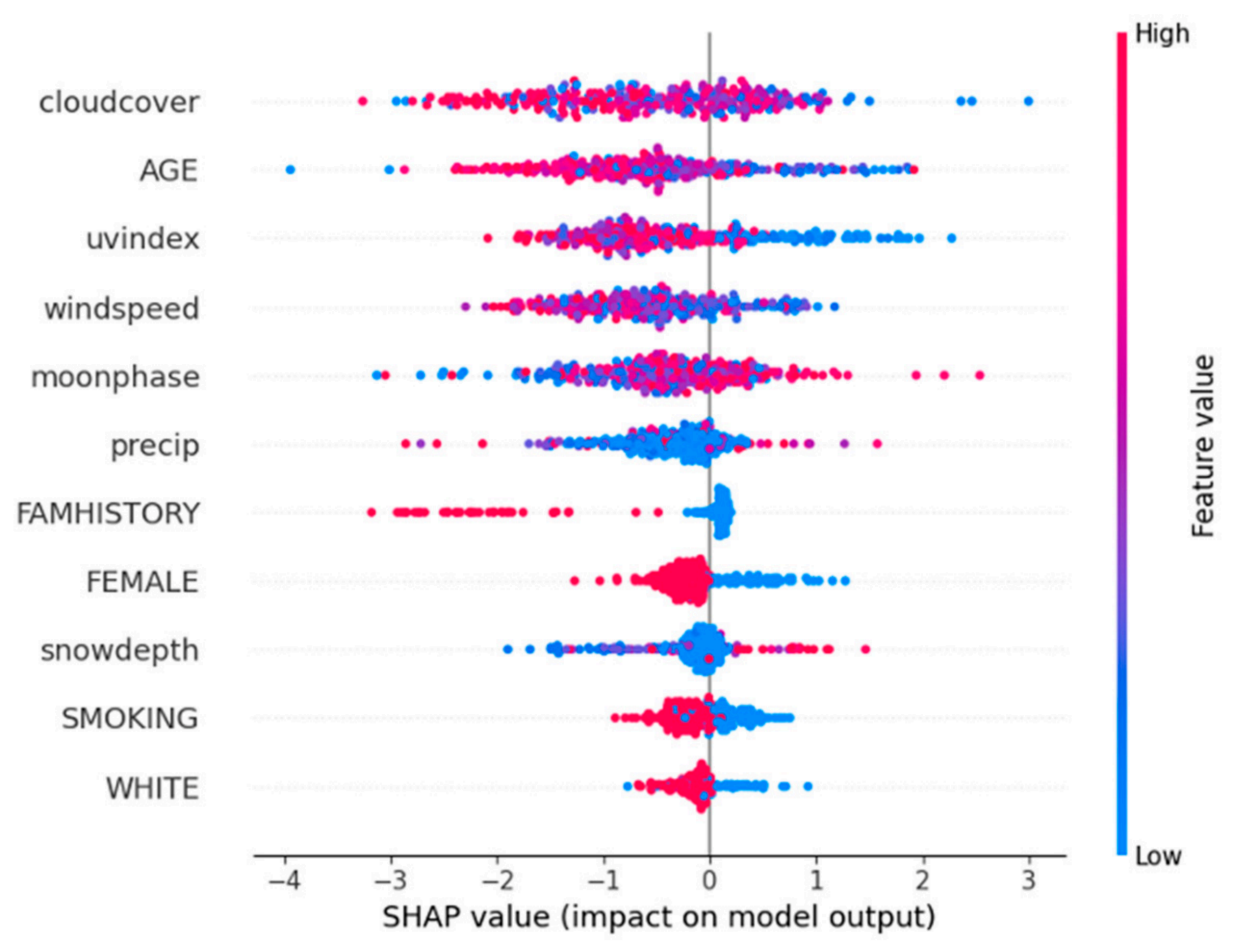
SHAP summary plot. This plot ranks all input variables by their overall impact on model predictions. Each horizontal strip represents one variable, with dots showing individual patient contributions. The x-axis is the SHAP value (positive = increased rupture prediction; negative = decreased prediction). The color gradient reflects the original value of the feature: red for high and blue for low. For example, red dots on the left side for “cloudcover” suggest that high cloud cover values generally decrease rupture risk, while blue dots on the right for “age” suggest that lower age tends to push the model toward predicting rupture.

**Figure 5:**
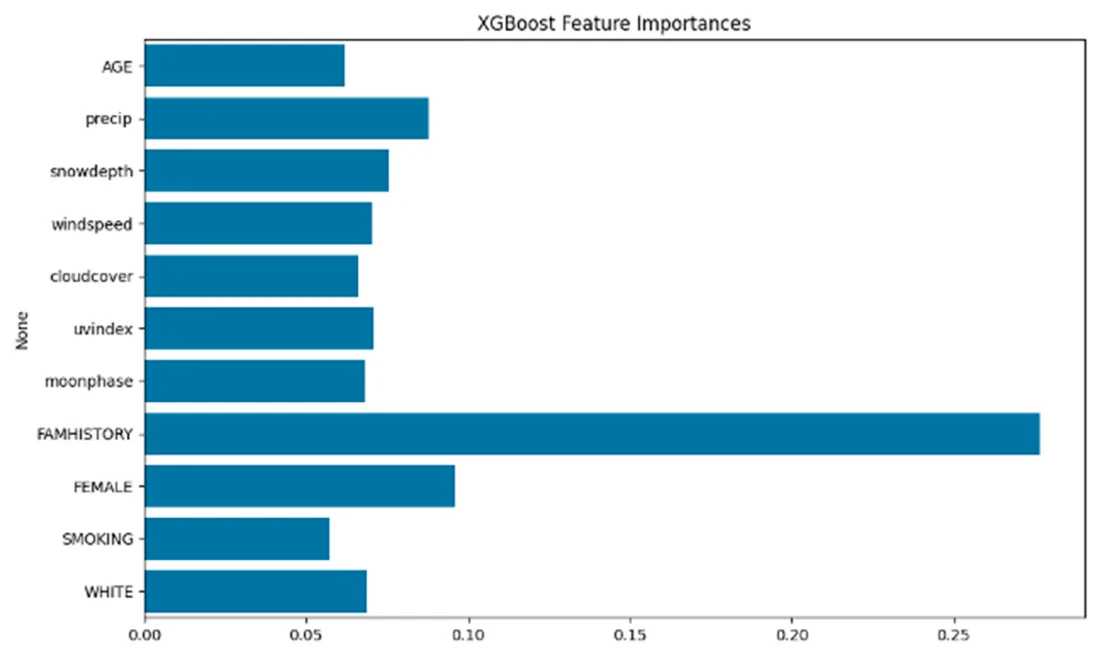
XGBoost feature importances in the machine learning model. Horizontal bars show the relative importance of each feature in the composite model, scaled so that all values sum to 1. Longer bars indicate a greater contribution to model predictions. Variable names appear as coded in the dataset (precip, precipitation; FAMHISTORY, family history of aneurysm; WHITE, White race).

Incorporating demographic variables, including age, sex, race, smoking history, and family history, modestly improved model performance. The composite model had an AUC of 0.590, an F2 score of 0.629, and a recall of 83%, with precision remaining similar at 32% and accuracy improving slightly to 49% (Figure 3). The corresponding SHAP summary plot for the composite model is shown in Figure 4. Feature importance analysis identified family history and female sex as the most important factors, with precipitation being the most important meteorological variable included (Figure 5).

To assess potential effect modification among features contributing to aneurysm rupture risk, we generated SHAP dependence plots using each feature as the primary variable. We overlaid SHAP values colored by relevant modifiers. SHAP dependence analysis revealed differential effects on model prediction based on predictor value and effect modification across multiple factors. Lower cloud cover was associated with more positive SHAP values (guiding the model toward rupture prediction), and this pattern varied with precipitation, snow depth, and UV index. SHAP values for moon phase were highest at values above 0.9, corresponding to the late waning phase approaching the new moon (on the 0 to 1 scale used by the weather dataset, in which 0.5 denotes the full moon), revealing a sharp increase in predicted rupture risk. This relationship was also more pronounced among smokers and subgroups with higher snow depth and precipitation. Age was consistently protective against rupture, and this effect was more pronounced in females. Smoking increased the risk of ruptured aneurysms across multiple meteorological variables.

## 4. Discussion

Several weather variables showed associations with rupture in univariate logistic regression, but only female sex remained significant, with sea level pressure trending toward significance. The final model’s low sensitivity suggests that weather variables provide limited predictive value for rupture in this population of endovascularly treated aneurysms. In contrast to prior nationwide and global studies of seasonal aSAH variation, this analysis focused on temperate-climate centers with similar healthcare infrastructures [9,21]. While hypertension, smoking, and other vascular risk factors are established contributors to aSAH, meteorological influences appear to have a modest effect in our study [22].

### 4.1. Meteorological Associations and Seasonal Variation

A decline in barometric pressure trended toward higher rupture risk in the complete-case model, though this trend did not persist in the full-cohort sensitivity analysis. Prior studies from the United States and Europe reported no association between barometric pressure and the risk of aneurysm rupture [23,24,25]. Short-term pressure drops exceeding 10 hPa over 24 h may be more relevant than absolute levels [26,27]. One case of aSAH during air travel suggested that rapid pressure changes can induce hypoxia, cerebral vasodilation, and increased perfusion, raising shear stress [28]. The occurrence of pressure drops in colder months may explain the seasonal variation in aSAH [8].

UV index also showed a univariate association with rupture, though effects may be confounded by pressure fluctuations [5,7]. These variables are strongly season-dependent. Prior work has yielded mixed results, but most studies have identified at least one seasonal factor [10,29,30]. Some studies have found a 35% higher risk of aSAH following an 8 °C temperature drop over two days [7,31]. Data from East Asia also showed winter peaks and spring declines [32]. These findings support a consistent seasonal pattern, likely mediated by vasoconstriction and sympathetic activation, which raise systemic resistance and perfusion, thereby stressing aneurysmal walls.

### 4.2. Predictive Modeling and Interpretability

Our machine learning analysis confirmed that meteorological variables alone have limited predictive value for aneurysm rupture. The weather-only model achieved relatively high recall but poor precision and accuracy, consistent with prior epidemiologic reports [9,21,33,34]. Even with the inclusion of demographic variables, precipitation, snow depth, wind speed, cloud cover, UV index, and moon phase all retained feature importance. Incorporating weather data has improved predictive performance in other machine learning models. For instance, adding weather dynamics to insurance claim triage increased cost-effectiveness by 6–9% [35]. Other studies have shown that models integrating weather and temporal factors can reliably forecast emergency medical services (EMS) demand, demonstrating that weather can influence acute event probabilities and resource needs, although not in isolation [36].

SHAP summary plots highlighted cloud cover and humidity as influential, although their effects varied by context. This variability may help explain inconsistent conclusions across observational studies [9,30]. Dependence analyses suggested that precipitation and snowfall modified the impact of cloud cover, with heavy snow correlating with reduced rupture prediction, possibly reflecting lower hospital attendance during storms rather than direct protection. Other studies, such as those in Canada, have found that the addition of snowfall improved a model for predicting patient emergency room (ER) attendance [37]. Additionally, SHAP identified established risk factors such as smoking as consistent contributors to model predictions [38,39], whereas UV index contributed less consistently and has not been reliably associated with rupture in prior studies [33,34]. While weather alone is likely insufficient to build a high-performing prediction tool, combining it with patient-level information in a machine learning framework such as XGBoost offers modest predictive improvement.

### 4.3. Strengths and Limitations

This study contributes contemporary, multicenter data to the literature examining potential meteorological contributions to aSAH. Our integration of meteorological data with clinical outcomes provides insight into potential environmental triggers of aSAH. However, our study is limited by its retrospective design, which prevents us from drawing causal conclusions. Weather exposure was assigned using same-day data at the date of admission, which may not capture preceding meteorological changes or the exact time of ictus; for ruptured aneurysms, admission may follow the ictus, and for unruptured aneurysms, the admission date reflects elective or incidental presentation rather than an acute event, limiting the comparability of exposure windows. Whether an aneurysm was known prior to the index admission was also not captured in the registry. In addition, hypertension, aneurysm size, and aneurysm location were not available for analysis; these are established rupture-risk factors, and their absence represents potential unmeasured confounding. Observations represent aneurysm cases rather than unique patients, and a subset of individuals contributed more than one case, including cases in both rupture groups; this non-independence was not modeled. While we assessed daily meteorological variables and demographic information, such as age and sex, future studies should examine genetic predispositions and aneurysm morphology, size, and location, which may be more prone to season-influenced rupture. Although SHAP analysis enhances the interpretability of XGBoost results, XGBoost remains a predictive rather than causal model; SHAP describes contributions to model predictions but does not establish causality, and the potential for model overfitting is an additional concern. SHAP can visually identify pairwise interactions, but it lacks built-in statistical tests to determine whether they are significant or reproducible.

Prospective studies that monitor potential blood pressure fluctuations, hormonal changes, and cerebral hemodynamic alterations should provide more causal and mechanistic insights. Expanding this research across various climates and populations would enhance generalizability beyond the climate of our study sites.

## 5. Conclusions

In this multicenter analysis, weather-related variables showed minimal predictive value for aneurysm rupture. Despite prior studies suggesting seasonal or atmospheric influences, our results indicate that meteorological factors have a limited association with rupture risk in Upstate New York. Weather on the day of presentation was not significantly associated with rupture, and the potential influence of preceding meteorological changes warrants further investigation; established patient-level risk factors remain the mainstay of surveillance and management.

## Figures and Tables

**Table 1:** Baseline characteristics of ruptured and unruptured intracranial aneurysm cases.

Characteristic	Ruptured (*n* = 377)	Unruptured (*n* = 1127)	*p*-Value
Age, years, mean ± SD	57.7 ± 13.7	59.5 ± 13.9	0.0279
Female, *n* (%)	245 (65.0%)	867 (76.9%)	<0.0001
Treatment center, *n* (%)			<0.0001
Albany Medical Center	258 (68.4%)	392 (34.8%)	
University at Buffalo	119 (31.6%)	735 (65.2%)	
Treated, *n* (%)	377 (100.0%)	1074 (95.3%)	<0.0001

**Table 2:** Univariate logistic regression results used for variable screening.

	Odds Ratio	95% Confidence Interval	*p*-Value
Temperature	0.999	0.992–1.005	0.748
Max Temperature	1.001	0.995–1.007	0.815
Minimum Temperature	0.997	0.990–1.003	0.347
Feels like Max	1.001	0.996–1.006	0.725
Feels like Min	0.998	0.992–1.003	0.425
Feels like	1.000	0.994–1.005	0.907
**Humidity**	0.988 **	**0.979–0.998**	**0.0135**
Precipitation	0.722	0.456–1.146	0.167
Precipitation Probability	0.999	0.996–1.001	0.310
Precipitation Coverage	0.995 *	0.989–1.000	0.0735
Snow	1.191	0.855–1.658	0.301
**Snow Depth**	1.360 **	**1.068–1.731**	**0.0126**
Wind Gust	0.994	0.981–1.007	0.344
Wind Speed	0.979 *	0.958–1.000	0.0532
Wind Direction	1.000	0.998–1.001	0.707
Sea Level Pressure	1.010	0.994–1.026	0.218
Cloud Coverage	0.998	0.994–1.003	0.536
Solar Radiation	0.999 *	0.997–1.000	0.0647
Solar Energy	0.986 *	0.971–1.001	0.0642
**UV Index**	0.951 **	**0.914–0.990**	**0.0146**
Dew	0.997	0.990–1.003	0.321
**Severe Risk**	1.016 **	**1.001–1.031**	**0.0403**
**Moon Phase**	1.589 **	**1.064–2.373**	**0.0237**

* *p* < 0.10; ** *p* < 0.05. Bold indicates *p* < 0.05.

**Table 3:** Multivariable logistic regression model.

	Odds Ratio	95% Confidence Interval	*p*-Value
**Age**	0.986 *	0.971–1.000	0.0573
**Sex**			
Male		Reference	
Female	0.605 **	**0.386–0.949**	**0.0285**
**Weather Metrics**			
Max Temperature	1.008	0.989–1.027	0.430
Precipitation	0.887	0.301–2.612	0.828
Precipitation Probability	0.999	0.993–1.005	0.770
Precipitation Coverage	0.991	0.971–1.010	0.347
Snow	1.184	0.670–2.092	0.562
Snow Depth	1.593	0.737–3.444	0.236
Wind Gust	1.009	0.976–1.044	0.594
Wind Speed	0.967	0.907–1.029	0.290
Wind Direction	1.000	0.997–1.002	0.797
Sea Level Pressure	0.962 *	0.926–1.000	0.0523
Cloud Coverage	1.002	0.989–1.015	0.727
Visibility	1.116	0.898–1.387	0.320
UV Index	0.928	0.821–1.049	0.234
Severe Risk	1.013	0.996–1.031	0.128
Moon Phase	1.225	0.582–2.578	0.592

* *p* < 0.10; ** *p* < 0.05. Bold values indicate *p* < 0.05. Bold row labels mark variable categories.

## Data Availability

The data presented in this study are available on request from the corresponding author due to privacy and institutional restrictions.

## Abbreviations

aSAH	Aneurysmal subarachnoid hemorrhage
OR	Odds ratio
CI	Confidence interval
VIF	Variance inflation factor
AUC	Area under the receiver operating characteristic curve
SHAP	SHapley Additive exPlanations
UV	Ultraviolet
XGBoost	Extreme Gradient Boosting
RIA	Ruptured intracranial aneurysm
ROC	Receiver operating characteristic
NOAA	National Oceanic and Atmospheric Administration
EMS	Emergency medical services
ER	Emergency room
IRB	Institutional Review Board
SD	Standard deviation
